# Green Synthesis of Gold Nanoflowers Using *Rosmarinus officinalis* and *Helichrysum italicum* Extracts: Comparative Studies of Their Antimicrobial and Antibiofilm Activities

**DOI:** 10.3390/antibiotics11111466

**Published:** 2022-10-25

**Authors:** Nurhan Ertas Onmaz, Dilek Demirezen Yilmaz, Kálmán Imre, Adriana Morar, Candan Gungor, Seyda Yilmaz, Dursun Alp Gundog, Adalet Dishan, Viorel Herman, Guven Gungor

**Affiliations:** 1 Department of Veterinary Public Health, Faculty of Veterinary Medicine, Erciyes University, Kayseri 38039, Turkey; 2 Department of Biology, Faculty of Science, Erciyes University, Kayseri 38039, Turkey; 3 Department of Animal Production and Veterinary Public Health, Faculty of Veterinary Medicine, University of Life Sciences “King Michael I” from Timișoara, 300645 Timisoara, Romania; 4 NanoBiotech R&D Company, Erciyes Teknopark, Kayseri 38030, Turkey; 5 Department of Biometrics, Faculty of Veterinary Medicine, Erciyes University, Kayseri 38039, Turkey

**Keywords:** antimicrobial activity, biofilms inhibition, *Helichrysum italicum*, gold nanoflower, *Rosmarinus officinalis*

## Abstract

This study was concerned with the green synthesis of gold nanoflowers (AuNFs) using the bioactive constituents of *Rosmarinus officinalis* (rosemary) and *Helichrysum italicum* (immortelle) extracts, as reducer and stabilizer agents along with the determination of their antibacterial and antibiofilm activity against *E. coli*, *S. aureus*, and *S. epidermidis*. The AuNFs were characterized using STEM, UV–Vis, DLS, ZETA, FESEM-EDX, and FTIR techniques. The antibacterial and antibiofilm activity of the AuNFs were evaluated by microdilution broth and microtiter plate (MTP) tests, respectively. STEM and DLS analysis confirmed the flower-like morphology of gold nanoparticle clusters of *R. officinalis*-AuNFs (R-AuNFs) and *H. italicum*-AuNFs (H-AuNFs) with a size of 20–130 nm and 15–90 nm, respectively. The MICs of R-AuNFs were found to be 40 µg/mL for *E. coli* and *S. epidermidis* and 160 µg/mL for *S. aureus*. The MICs of H-AuNFs against all bacterial strains were 20 µg/mL. All tested AuNFs exhibited a strong dose-dependent antibiofilm activity against the test strains, and H-AuNFs was more effective than R-AuNFs. The green synthesis of AuNFs from the rosemary and immortelle extracts can be applied as a potential agent to overcome the growth of biofilm-producing microorganisms in food industries.

## 1. Introduction

The rapid emergence and dissemination of resistant bacteria constitute one of the most serious global threats to human and animal health, food safety and security, and economic and agricultural development [1,2,3]. In addition to that, the biofilm-forming ability (BFA) of bacteria protects themselves from host immunity, antibiotics, and disinfectants, which make them a major cause of persistent and recurrent infections [4,5,6,7]. For that reason, it has become increasingly important to develop a new, powerful therapeutic approach to eradicate and control resistant pathogens [8,9].

The recent developments in nanotechnology have allowed the utilization of nanoparticles to be used as biological agents for inhibiting microorganisms [6,10,11,12,13]. Nanoparticles are small particles ranging from 1 to 100 nm in size and have high surface energy. These unique properties make them suitable for a number of industrial fields, such as chemical, pharmaceutical, mechanical, and food processing industries [14,15]. Silver, gold, platinum, nickel, manganese, titanium, and zinc are metal-based nanoparticles that have non-toxic and antimicrobial activities [11,16]. Among the metal-based NPs, gold nanoparticles (AuNPs) are especially encouraging for their applicability in the biological domains, due to their unique surface plasmon resonance properties, easy synthesis, adjustable sizes, and multifunctional capacity with well-characterized features [11,16,17]. Different methods based on physical, chemical, biological, and hybrid techniques can be used for the synthesis of NPs. Physical and chemical methods have some problems, such as being expensive, using toxic chemicals as a major constituent, and having complex equipment and synthesis conditions [18,19].

Recently, the green synthesis methods of nanoparticles are drawing considerable attention as an alternative approach to conventional chemical and physical methods in terms of their ecofriendliness, cost-effectiveness, safe handling, biocompatibility, and possessing a broad variability of metabolites that have antioxidant and antimicrobial activities [6,17,20,21,22]. In this regard, bacteria (*Bacillus marisflavi, Pseudomonas putida*, *Enterobacter cloacae* Z0206, *Citrobacter freundii* Y9, etc.), fungi (*Trichoderma reesei*, *Fusairum oxysporum*, etc.), algae (*Ascoseira mirabilis*, etc.), and plant extracts (*Centella asiatica*, *Syzium cumini*, *Plumeria alba*, etc.) are used as alternative reagents to chemical agents in the synthesis of nanoparticles [6,14,16,17,21,22,23,24,25]. The green methods, especially plant-mediated methods, lead to easy biosynthesis of flower-like gold nanoparticles (also referred to as gold nanoflowers–AuNFs). Here, green materials can play a dual role, by acting as both reducer and stabilizer for the synthesis of AuNFs. Firstly, biomolecules from green materials reduce Au (III) to Au (0), then NPs can be stabilized through agglomeration, and finally, AuNFs are formed [11,26,27,28]. The advantages of green synthesis techniques are the controllable shape and size of AuNFs [27,28].

Medicinal plants and their derivatives are attracting great interest due to their high efficiency, low toxicity, and different pharmacological properties [29,30]. There are many reports on the synthesis of AuNPs using several plant-based extracts, including *Magnolia kobus* [31], *Plumeria alba* [32,33], *Carica papaya* and *Catharanthus roseus* [34], *Syzygium cumini* [24,35], and *Acanthopanacis cortex* [36]. These studies have suggested that AuNFs are non-toxic, in comparison with other metallic NPs [24,33,36]. Among the medicinal plants, *Helichrysum italicum* (immortelle; family Asteraceae) and *Rosmarinus officinalis* (rosemary; family Laminacae) are naturally occurring in the Mediterranean region and are widely used in traditional and folk medicine (especially respiratory, digestive, and skin inflammatory conditions) for their anti-inflammatory, antibacterial, antioxidant, antiallergic, and dermofunctional properties [37,38,39,40]. Due to these properties, they are also employed in the cosmetic and food sectors [41,42,43,44,45].

Numerous studies have documented the antimicrobial activity of *H. italicum* [30,40,46] and *R. officinalis* extracts [47,48,49]. To our knowledge, a few recent studies have also focused on nanoparticle synthesis using rosemary extracts as reductants [20,50,51], yet there is no literature knowledge about nanoparticle synthesis from immortelle extracts. Therefore, this study was designed for the green synthesis of flower-like AuNPs, using *H. italicum* and *R. officinalis* plant extracts as reducing agent, and to investigate their antimicrobial and antibiofilm activities against biofilm-forming model reference strains (*E. coli* ATCC 35218, *S. epidermidis* ATCC 35984, and *S. aureus* ATCC 25923).

## 2. Results

**Figure 1:**
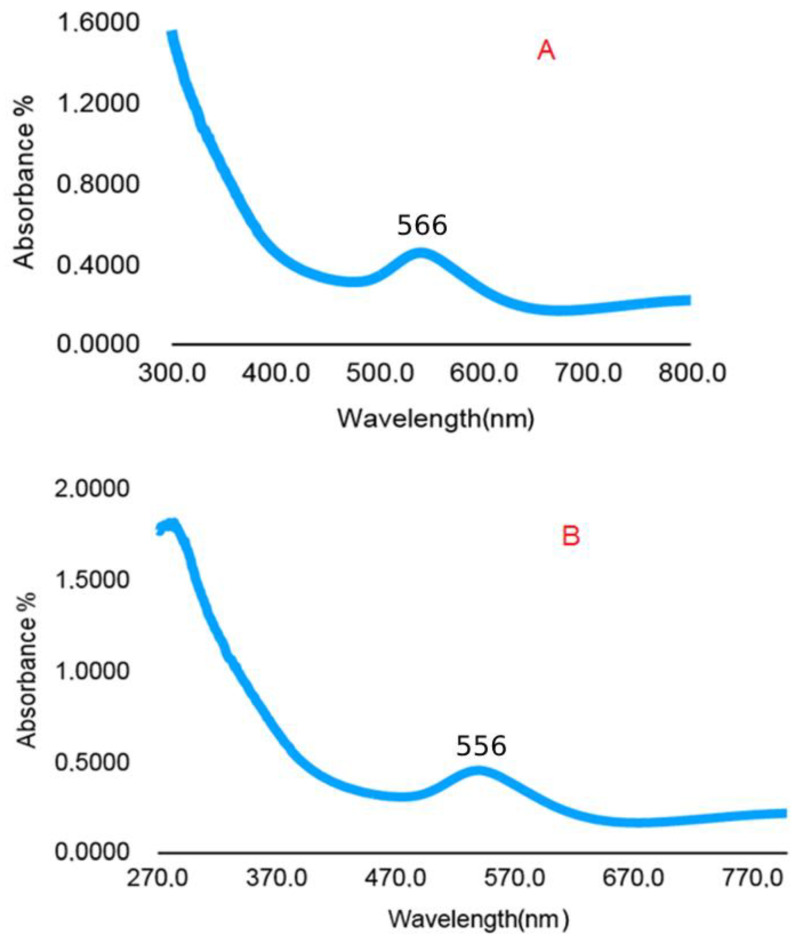
UV–Vis adsorption spectra of AuNFs. (**A**) H-AuNFs absorbance picks from 566 nm; and (**B**) R-AuNFs absorbance picks from 556 nm.

**Figure 2:**
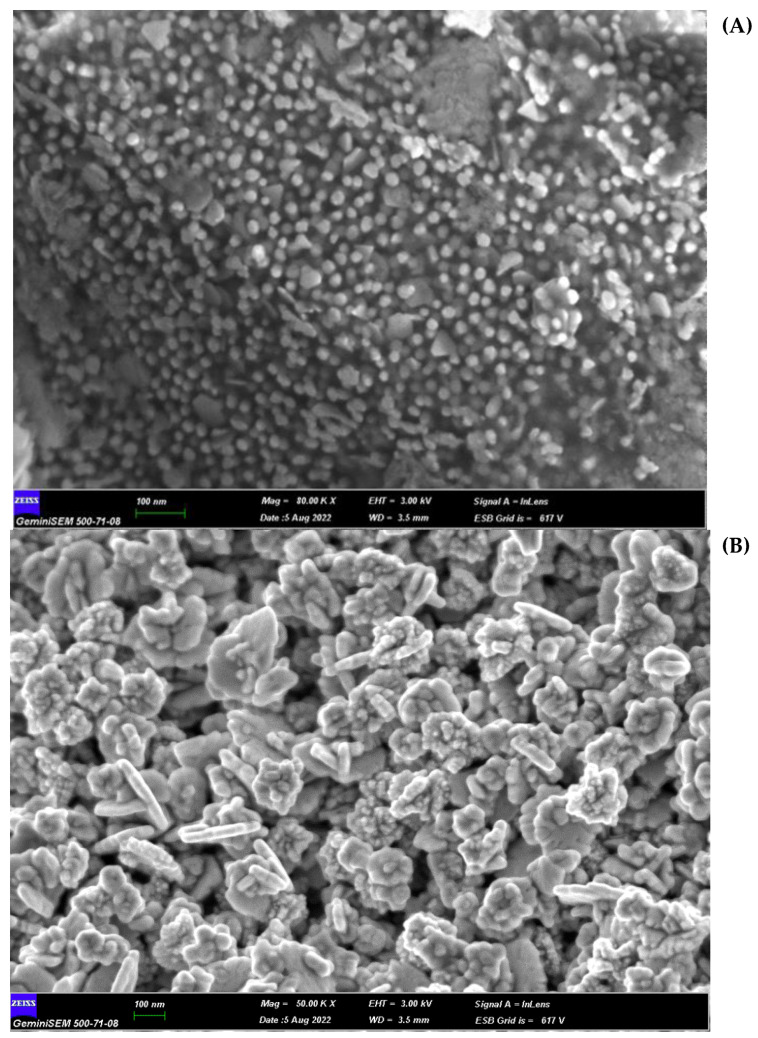
FE-SEM image showing the surface morphology of green synthesized AuNFs. AuNFs represent gold nanoflowers; H-AuNFs from *Helichrysum italicum* extract (**A**); and R-AuNFs from *Rosmarinus officinalis* extract (**B**).

**Figure 3:**
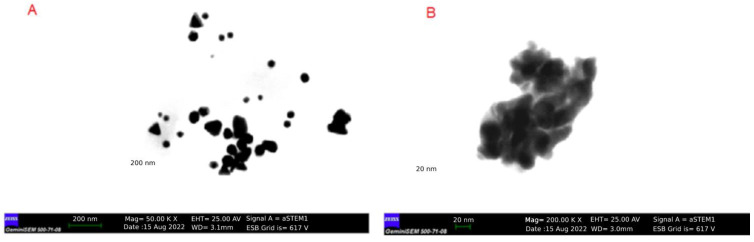
STEM image of H-AuNFs showing the particle shapes. (**A**) Dispersed H-AuNFs (bar scale: 200 nm); (**B**) magnified image (bar scale: 20 nm).

**Figure 4:**
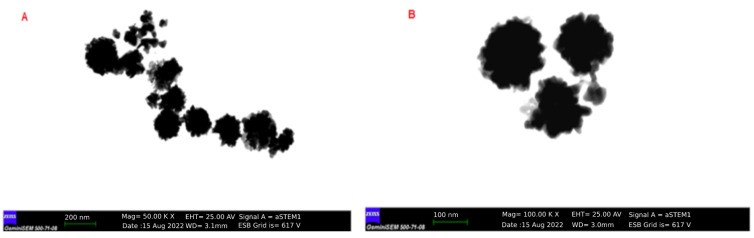
STEM image of R-AuNFs showing the particle shapes. (**A**) Dispersed R-AuNFs (bar scale: 200 nm); (**B**) magnified image (bar scale: 100 nm).

As an indicator of the reduction of HAuCl_4_·3H_2_O to gold nanoparticles, for the *H. italicum* extract, the change in color of the H-AuNFs reaction mixture was from yellow to deep purple; for the *R. officinalis* extract, the color of the R-AuNFs mixture changed from yellow to green. The synthesis of AuNPs was verified by UV–Vis absorption spectra. The UV–Vis spectra of the AuNFs exhibited a surface plasmon resonance (SPR) band at 566 nm and 556 nm, respectively, for H-AuNFs and R-AuNFs (Figure 1), consistent with the formation of AuNFs. STEM and FE-SEM analysis confirmed that AuNPs were flower-shaped (Figure 2, Figure 3 and Figure 4).

The STEM image indicated that the synthesized AuNFs were observed to be small particles with nearly spherical shapes (1–10 nm size). In detail, H-AuNFs were characterized as poly-dispersed, with triangle, pentagon, and nearly spherical shapes, with the size ranging from 15 to 90 nm (69 nm average). R-AuNFs were nearly spherical in shape, with the size ranging from 20 to 130 nm (120 nm average). In addition, AuNFs exhibited separate petal-like shapes in a flower from the higher magnification STEM images up to 20 nm (Figure 3 and Figure 4).

**Figure 5:**
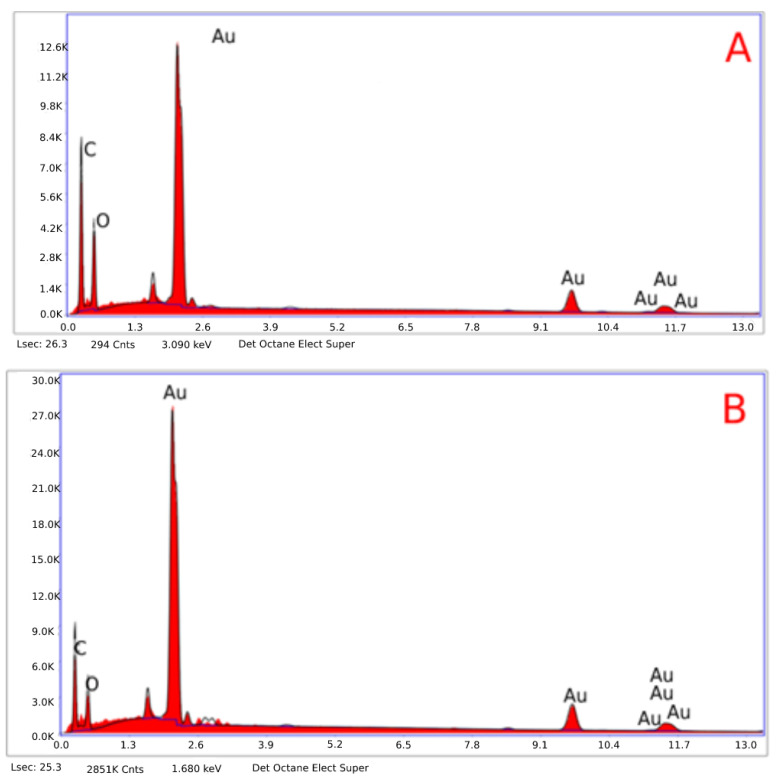
EDX spectrum shows a sharp peak at 2.3 keV confirming gold’s presence. (**A**) H-AuNFs and (**B**) R-AuNFs.

The EDX spectrometer spectrum obtained from FE-SEM indicated that the obtained AuNFs were pure (Figure 5a,b). Additionally, elemental mapping analysis showed the maximum distribution of gold elements. The hydrodynamic diameter of the H-AuNFs and R-AuNFs were 69 and 160 nm, respectively, according to DLS analysis results. In addition, H-AuNFs and R-AuNFs had a negative zeta potential, indicating their high stability, with a charge of −21 and −27 mV, respectively.

**Figure 6:**
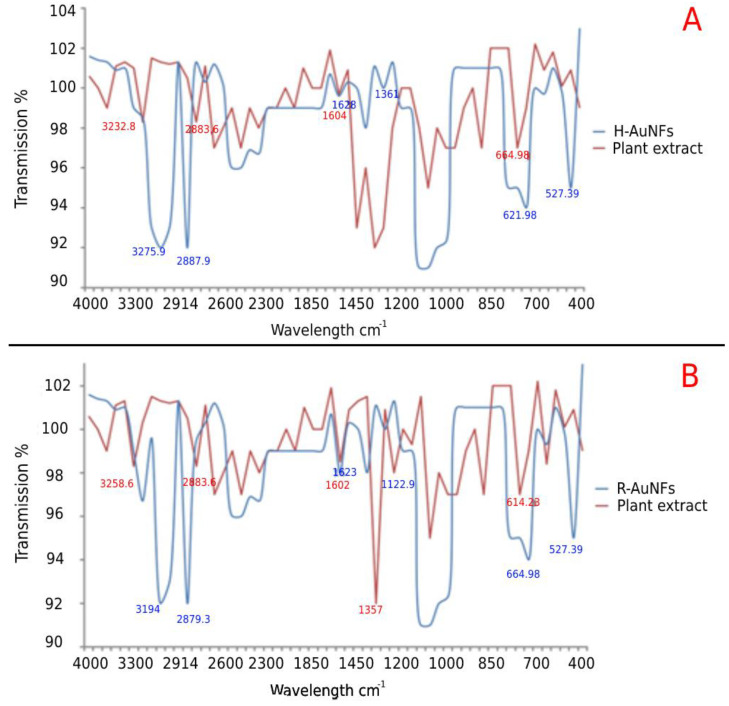
FTIR analysis of AuNFs and plant extracts. AuNFs represent gold nanoflowers; H-AuNFs from Helichrysum italicum extract (**A**); and R-AuNFs from Rosmarinus officinalis extract (**B**).

The FTIR spectra of the AuNFs are shown in Figure 6. For comparison, the spectra of plant extracts were also presented. As shown in Figure 6, the synthesized AuNFs generally had 4 to 6 prominent peaks in the FTIR spectra.

In detail, the *H. italicum* plant extract showed five main absorption peaks at 3232.8–505.89 cm^−1^, and H-AuNFs exhibited six intense peaks at 3275.9–527.39 cm^−1^, indicating the successful synthesis of H-AuNFs (Figure 6). The broad peaks at 3275.9, 12,887.9, and 1628.1 cm^−1^ corresponded to the presence of the -OH group and the C–H stretching of alkanes and stretching vibrations of the −C− group, respectively. In addition, six intense bands were observed at 3258.6–614.23 for *R. officinalis* extract, as well as four broad bands at 3194.0–664.98 cm^−1^ for R-AuNFs (Figure 6). The FTIR spectrum of R-AuNFs showed that the absorbance peak at 3194 cm^−1^ corresponds to the N–H group. Similar to the FTIR results of H-AuNFs, the peak at 1 and 2879.3 and 1623.8 cm^−1^ indicated C–H stretching of alkanes and stretching vibrations of the −C− group, respectively. Some peak intensities are reduced after nanoparticle production because they may be used as a capping agent. These results showed that the same compounds were responsible for both nano flowers production.

**Figure 7:**
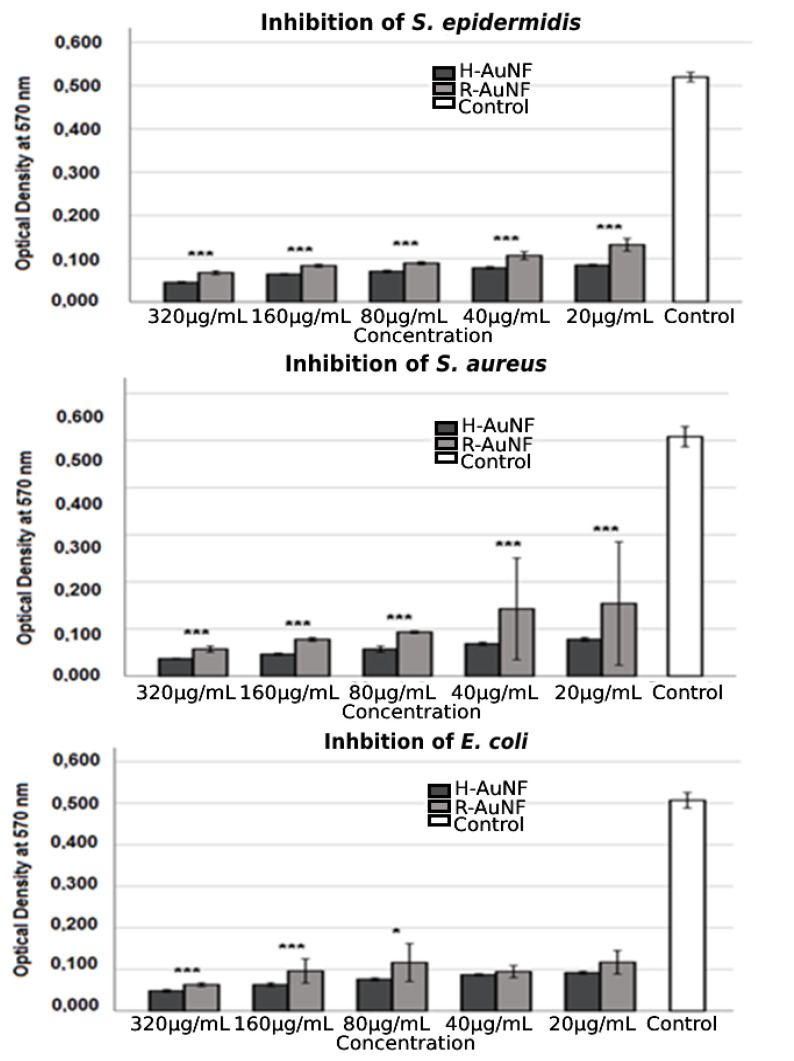
Antibacterial activity of synthesized AuNFs against selected bacteria strains at different concentrations. The error bars symbolized the SD of error mean (* *p* < 0.05; *** *p* < 0.001).

H-AuNFs and R-AuNFs exhibited a strong concentration-dependent antimicrobial activity against all model bacterial strains included in the study. The results revealed that more potent inhibitory activity was observed when the concentration of AuNFs was increased. Noteworthy, H-AuNFs exhibited high antibacterial activity with MICs up to 20 μg/mL and MBC at 20 μg /mL as compared to R-AuNFs in all test strains (*p* < 0.05). The MIC and MBC values of R-AuNFs against *E. coli*, *S. epidermidis*, and *S. aureus* were 40, 40, 160, and 80, 80, 320 µg/mL, respectively (Table 1). It was noted that there is no statistically detected difference between low concentrations (40 µg/mL and 20 µg/mL) of AuNFs regarding the inhibition of *E. coli* growth in our study (Figure 7). However, in further studies performed to evaluate the antibacterial activity of rosemary and immortelle extracts, the MICs of both plant extracts were recorded as > 320 µg/mL.

**Figure 8:**
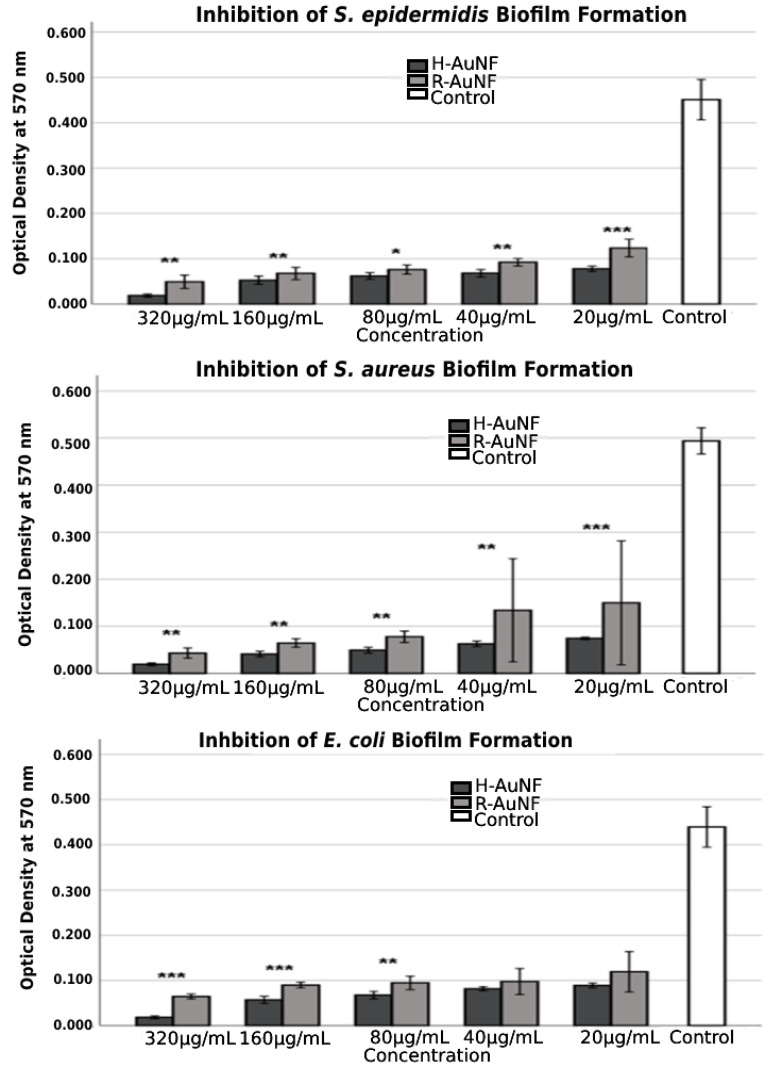
Biofilm inhibitory activity of AuNFs against selected bacteria strains at various concentrations. The error bars symbolized the SD of error mean (* *p* < 0.05; ** *p* < 0.01; *** *p* <0.001).

According to the obtained MTP results, the antibiofilm activity of the tested AuNFs were dependent on their concentrations. As shown in Figure 8, both AuNFs successfully inhibited the biofilm formation of test strains with inhibition percentages ranging from 70 to 96% compared to the control samples without AuNFs; however, H-AuNFs were more effective than R-AuNFs with statistically significant differences (*p* < 0.05).

In detail, in the presence of 320 µg/mL concentrations of AuNFs, the maximum prevention rates against biofilms of *S. aureus*, *S. epidermidis*, and *E. coli* were 96% for H-AuNFs, and 91.2%, 89.1%, and 85.2% for R-AuNFs, respectively. At 20 µg/mL, the AuNFs also notably reduced the biofilm formation of *S. aureus*, *S. epidermidis*, and *E. coli*, in which percentages of biofilm inhibitions were 84.9%, 82.7%, and 79.7% for H-AuNFs and 69.6%, 72.5 %, and 72.8 % for R-AuNFs, respectively. In addition, the highest inhibition was obtained for *S. aureus* at 320 µg/mL concentrations of both AuNFs, followed by *S. epidermidis* and *E. coli* (Table 2).

**Figure 9:**
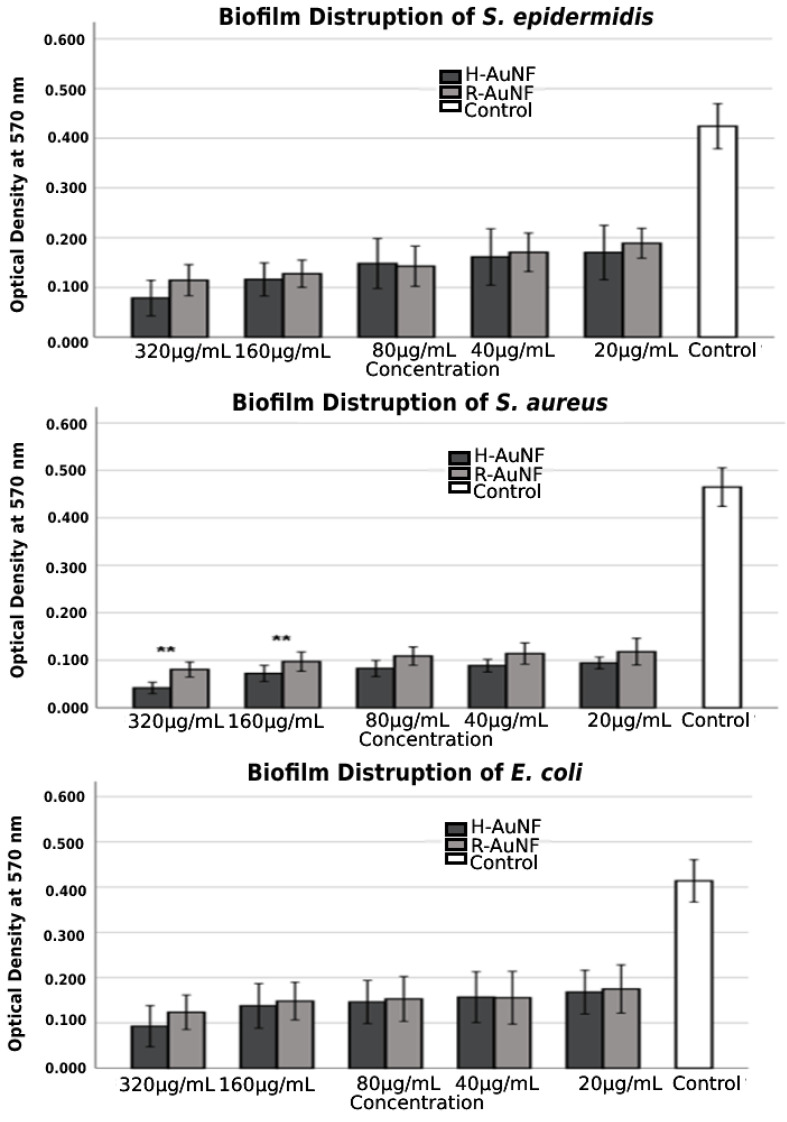
Biofilm disruption activity of AuNFs against selected bacteria strains at various concentrations. The error bars symbolized the SD of error mean (** *p* < 0.01).

Similarly, according to the obtained results, both AuNFs disrupted pre-formed biofilms in concentration-dependent manner (Figure 9), and the highest disruptive effect was recorded against *S. aureus* for both AuNFs. No significant difference in biofilm eradication was observed among AuNFs for *S. epidermidis* and *E. coli* at all tested concentrations (*p* > 0.005). For *S. aureus*, H-AuNFs were more successful at disrupting pre-formed mature biofilm by 91% and 84.4% at 320 µg/mL and 160 µg/mL concentrations, respectively, in comparison with R-AuNFs, as shown in Table 2 (*p* < 0.005). The morphological changes in biofilm cells after AuNFs treatment were visualized with SEM. According to findings of SEM analysis, the multitiered biofilm growth was seen in the control samples without AuNFs. In contrast, the treatment group with both AuNFs exhibited a significant reduction in the intensive aggregation of all the test bacterial cells, with increasing concentration (Supplementary Figures S1–S3).

## 3. Discussion

In the present study, *Rosmarinus officinalis* leaf and *Helichrysum italicum* flower extracts were utilized as reducing and stabilizing agents. The aqueous gold ions were reduced during the exposure to these plant extracts, and thus synthesis and stabilization of AuNFs were provided. The reaction mixture’s color change, from yellow to deep purple for H-AuNFs and from yellow to green for R-AuNFs, was the first evidence of the formation of the AuNFs. These findings were in agreement with a previous study conducted by Dzimitrowicz et al. [52] about AuNP synthesis from *R. officinalis*, but there are no reports on gold nanoparticle synthesis in *H. italicum*. The colors observed in this study are a result of the absorption or scattering of light that passes through the gold particles [24,53]. It was previously reported that AuNPs can appear red, purple, blue, or other colors depending on their size, shape, and amount of aggregation due to surface plasmon resonances [24,53,54,55,56].

In our study, the color of the reaction mixture changed within 5 to 7 min and this reaction did not require input energy; thus, it represents an economic and ecological approach to the synthesis of AuNFs [57,58]. Numerous studies have also reported the mechanism of formation of organic–inorganic hybrid nanoflowers formation [2,59,60,61,62].

UV–Vis spectroscopy is the most effective and simple technique to confirm the formation of nanoparticles via measuring the surface plasmon resonance (SPR), and gold nanoparticles generally show an SPR peak between 500 and 600 nm [63,64]. In this study, a similar pattern and peak with a value of 566 nm and 556 nm were recorded for H-AuNFs and R-AuNFs, respectively. In addition, the color change of the reaction mixtures of the AuNFs was coherent with the SPR peak for gold particles. Similarly, previous studies have reported that this type of broad peak is usually observed for branched nanoparticles, such as nanoflowers [65,66,67,68,69,70,71].

STEM image results demonstrated that H-AuNFs have a different morphology consisting of poly-dispersed, triangle, pentagon, and nearly spherical shapes, while R-AuNFs are nearly spherical. In the literature [72,73,74,75] spherical, triangular, and hexagonal gold nanoparticles have been reported; however, in this study, flower-shaped nanoparticles were also obtained in agreement with Borah et al. [24].

H-AuNFs demonstrated a slightly smaller particle size (average 69 nm) than those of R-AuNFs (average 120 nm), as the SPR peak of H-AuNFs exhibited a shorter wavelength compared with R-AuNFs. The reason for differences in the morphology of AuNFs might be attributed to the variability in the composition and amounts of reducing agents in the used plant extracts [52]. Plant extracts include numerous compounds (phenols, amines, ketones, aldehydes, terpenoids, polyols, alkaloids phenolic acids, proteins, etc.), which are responsible for the reduction and stabilization of nanoparticles [76,77].

The particle size of AuNFs were confirmed with particle hydrodynamic diameters, which were obtained using the DLS method. H-AuNFs yielded an average size diameter of 69 nm, consistent with that of their STEM result. However, the measured particle size of the R-AuNFs using STEM (average 120 nm) was smaller than that obtained with the DLS technique (average 160 nm). This discrepancy is possibly due to different sample conditions. DLS measures hydrodynamic sizes of particles in an aqueous solution, whereas STEM analyses are performed on dry samples. Therefore, particle size measured from DLS generally is larger than STEM [35,78].

The FTIR results of H-AuNFs and R-AuNFs suggested that phenolic compounds and carbonyl groups were responsible for the reduction of gold salt. Different biological molecules, such as carbonyl, alcohol, phenolic, and amines, from both plants have important roles as reducing, capping, and stabilizing agents in nanoparticle production. According to the obtained results, the C=N bands were also observed for H-AuNFs compared to the corresponding band of *H. italicum* plant extract. It could be concluded that, the gold nanoclusters from both plants are not naked and are embedded with some biogenic compounds from these plant samples. Similar reports were obtained by different studies in the literature [24,79,80]. Jiang et al. [79] stated that, water soluble biomolecules from different plants play important roles as reducing agents in nanoparticle synthesis. It was noted that AuNFs had good stability due to their negative zeta potential (−21 mV for H-AuNFs and −27 mV for R-AuNFs). The negative surface charge prevents possible aggregation by inducing charge repulsion between different nanoparticles, thus providing efficient and long-term stabilization [6,81].

To date, several studies have demonstrated the antimicrobial and antibiofim activity of different nanomaterials, such as silver, gold, copper, zinc, iron, and magnesium oxide [20,82,83,84,85,86,87,88,89]. Furthermore, gold nanomaterials have attracted tremendous attention in fighting against the pathogens due to unique properties such as size, shape, surface and optical properties, low toxicity, ease of synthesis, and high stability [21,81]. It has been reported that AuNFs are more effective and advantageous than conventional AuNPs in biological applications in several studies [67,87]. This advantage has been associated with better colloidal stability and larger surface area due to AuNFs’ multi-branched three-dimensional structure. Three-dimensional shaped nanoparticles hold the largest contact area to interact with the bacterial cell wall or genome, so it can easily cause structural cell deformation [24,87].

Our results clearly indicate that both H-AuNFs and R-AuNFs showed strong antibacterial activity against Gram-positive and Gram-negative bacteria. Similar results have been reported earlier on the antimicrobial effect of synthesized AuNPs using different plant extracts [24,34,81,90,91]. It has been stated that the antibacterial effectiveness of the nanoparticles depends on particle size, high surface area to volume ratio, and sample morphology [24,81,91,92]. A lot of hypotheses have been proposed to explain the inhibitory effect of gold nanoparticles on bacteria: (a) uptake of free gold ions followed by disruption of the cell membrane or cell walls; (b) enzyme inhibition; (c) disruption of energy metabolism and electrolyte balance; (d) inhibition of nucleic acid synthesis; (e) protein deactivation; and (f) reactive oxygen species (ROS) generation by AuNPs. The most accepted mechanism for the antibacterial activity of the gold nanoparticles is the oxidative stress of bacterial cells because it leads to the release of intracellular lactate dehydrogenase enzyme [44]. However, Lee and Lee [26] documented that AuNPs inhibit cell growth without directly causing membrane damage and they induce an imbalance in redox status without increasing ROS levels. Their result suggests that other mechanisms (such as membrane depolarization, severe DNA damage, caspase-like protein activation) might play a role in bacterial inhibition.

There was a positive correlation between their inhibitory effect with the increase in their concentration, and H-AuNFs displayed more effectiveness than that of R-AuNFs. It can be attributed that the bigger size and negative surface charge of R-AuNFs could cause these results. Small NPs have a better chance of entering the bacterial membrane due to their relatively larger surface-to-volume ratio, hence displaying stronger antimicrobial activity [24,81,88,91,93]. Furthermore, the antibacterial activity of both AuNFs was concentration-dependent similar to the earlier reports [89,91,94,95]. Bacterial cells underneath in the biofilm are 1000-fold more resistant to antibacterial agents than planktonic cells, hence the disruption of preformed mature biofilm is quite difficult [96]. Here, we clearly demonstrated that H-AuNFs and R-AuNFs, in a concentration-dependent manner, not only strongly inhibited biofilm formation but also eradicated preformed biofilms of test bacteria. In addition, SEM analysis suggested that both AuNFs easily interacted with bacterial cells embedded in a biofilm and resulted in biofilm disruption. Similar results on the antibiofilm effect of AuNPs were reported by several authors [6,97,98,99,100,101]. Biofilm inhibition could be due to the degradation of the EPS layer present in the biofilm, inhibition of bacterial adhesion, and suppression of genes related to biofilm formation. However, biofilm formation occurs in four major stages, including bacterial attachment to the surface, microcolony formation, biofilm maturation, and detachment of bacteria. The inhibition of one of these stages causes pathogens in biofilm to be vulnerable [101]. Therefore, the antibiofilm effect mechanism of AuNFs needs to be further studied, also genetically.

## 4. Materials and Methods

### 4.1. Materials

Analytical grade Tetrachloroauric (III) acid trihydrate (HAuCl_4_·3H_2_O) was purchased from Sigma-Aldrich Co. (St Louis, MO, USA). Plants were purchased from the local market in Türkiye and used after the biological identification by a plant taxonomist at the Department of Botany, Erciyes University, Turkey.

### 4.2. Bacterial Strains

The antibacterial effect of the AuNFs was tested against some important opportunistic pathogens that have biofilm formation ability, including *S. aureus* ATCC 25923, *S. epidermidis* ATCC 35984, and *E. coli* ATCC 35218. This strain was obtained from the American Type Culture Collection (ATCC) in the USA. All culture media used in this study were purchased from Merck (Darmstadt, Germany) and the test organisms were cultured under aerobic conditions at 37 °C for 24 h. The concentrations of the microorganisms were adjusted to 1 × 10^8^ CFU mL at 0.5 McFarland standard turbidity for all tests.

### 4.3. Methods

#### 4.3.1. Green Synthesis and Characterization of AuNFs

In this study, *H. italicum* and *R. officinalis* were used as organic parts for the AuNFs synthesis. Both plant extracts were prepared with distilled water in an individual glass beaker. About 25 g of ground powder of flowers of *H. italicum* and leaves of *R. officinalis* were extracted with distilled water maintaining 100 mL volume for 50 min at 80 °C to take out its aqueous extract. These aqueous extracts were collected by filtration to completely remove the particulates. A total of 4 mL of obtained extracts were mixed with ultra-pure distilled water for the synthesis of gold nanoparticles. HAuCl_4_·3H_2_O, at the final concentration of 1 mM, was added to each plant extract and stirred for 10 min at room temperature. At the end of the synthesis, the color of plant extracts was expected to change, which indicates the reduction of Au^3+^ to Au^0^. Afterward, a centrifuge was performed at 16,000 rpm for 25 min to collect the gold nanoparticles. Next, the supernatant was removed to eliminate unreacted gold ions. Finally, the obtained pellet was dried at 50 °C after washing several times with distilled water, and was used for further characterization and confirmation.

Morphological characterization of the synthesized AuNFs was performed with scanning transmission electron microscope (STEM) and field emission scanning electron microscopy (FE-SEM). The elemental composition of the samples was determined by energy dispersive X-ray analysis (EDX, ZEISS EVO LS10, Waltham, MA, USA). The presence of functional groups involved in the NF structure by Fourier transform infrared spectroscopy (FTIR, Perkin Elmer 400 FT-IR Spectrometer Spotlight 400 Imaging System, Waltham, MA, USA) analysis was detected. The UV-Vis Spectrophotometer was used to measure the absorption spectra of AuNFs. DLS data and zeta potential of AuNFs were obtained at 25 °C with a Horiba particle size analyzer.

#### 4.3.2. Antimicrobial Activity of AuNFs

The antimicrobial activity of the AuNFs against *S. aureus*, *S. epidermidis*, and *E. coli* was evaluated using broth micro-dilution techniques with Muller–Hinton broth (MHB) in the sterile 96-well round-bottomed polystyrene microtiter plates. A 100 μL of each stock of AuNFs was pipetted into the top row (A) of individual 96-well plates. To the other rows (B–G) 50 μL of MHB was added. Samples tested were diluted to a final concentration of 20 mg/mL in individual 96-well plates (from row B to row E) using a 2-fold serial dilution method. Rows of F containing tested bacteria and MHB without bacterial strains (row G) were positive and negative control, respectively. The bottom rows of plates included just AuNFs for contamination control. Next, 50 μL of each bacterial culture (~1 × 10^8^ cfu/mL) was added to triplicate wells of an individual 96-well plate, containing 50 μL of AuNFs at different concentrations. Each plate was covered loosely with cling film to prevent the bacteria from becoming dehydrated and incubated at 37 °C for 24 h. The MIC values of R-AuNFs and H-AuNFs were considered for each bacterial isolate as the lowest concentration of AuNFs inhibiting any visible bacterial growth after 24 h incubation. Following the MIC determination of AuNFs, a 10 µL aliquot of each well with the concentrations that indicated growth inhibition (clear) was subcultured to an MH agar and incubated for 24 h at 37 °C. This process was performed three times for the determination of minimum bactericidal concentration (MBC). After the incubation period, the lowest concentrations of inhibiting bacterial growth were noted [2]. Further, the antimicrobial activity of plant extracts was also made as above.

#### 4.3.3. Tolerance Level

The tolerance levels of each bacterial strain against AuNFs were calculated using the following formula, previously stated by May et al. [102]:

Tolerance = MBC/MIC

#### 4.3.4. Inhibitory Effect of AuNFs on Biofilm Formation

The inhibition effect of 32 to 256 μg/mL concentrations of synthesized AuNFs on the biofilm of the three bacterial strains tested was determined by a 96-well microplate assay as previously described [103,104]. Briefly, bacterial strains were inoculated in Trypticase Soy Broth (TSB) with 2 % glucose and incubated overnight at 37 °C. Following the incubation, the wells of each test plate were filled with 100 μL of TSB with 2 % glucose, then 100 μL of AuNFs (three wells for each concentration) at different concentrations (MIC to 1/16 MIC) and 100 μL of 0.5 McFarland test bacterial cultures. Negative control wells contained 100 μL of tested bacteria and 100 μL of TSB. The plate was incubated at 37 °C for 24 h. Next, the contents of the wells were carefully removed, washed three times with phosphate buffer (PBS, pH = 7.2), and air-dried. After that, biofilms were fixed with 200 μL of 99% (*v*/*v*) methanol for 15 min, and then methanol was removed, and the plates were left to dry. Each well was rinsed with tap water to remove the unbound dye, following the staining with 200 μL of 1% crystal violet dye for 5 min, and then the plates were air-dried. Finally, 200 μL of 33% glacial acetic acid was added to each well and incubated at 22 °C for 10 min to dissolve the CV stain and the absorbance at 600 nm was measured in a microplate reader (Thermo-Scientific, Waltham, MA, USA). Each assay was carried out twice with three replicates and the data are given as the mean ± SD. The percentage of biofilm inhibition was calculated by comparing the mean absorbances of the AuNFs-treated wells with that of the negative control (bacterial strains without AuNFs) with the following formula:

Inhibition % = (OD_600_ nm of negative control) − (OD_600_ nm of AuNF)/(OD_600_ nm of negative control) × 100

#### 4.3.5. Effect of AuNFs on Disruption of Preformed Biofilm

For this purpose, biofilms were formed by adding 100 μL of bacterial strains tested (1 × 10^6^ CFU/mL) into 96-well polystyrene microtiter plates containing 100 μL of TSB with 2% glucose. Plates were incubated at 37 °C for 24 h. After incubation, the contents of the wells were carefully aspirated, washed twice with PBS, and the plates were dried. Then, 100 µL of AuNFs (three wells for each concentration) at different concentrations (MIC to 1/16 MIC) were added into the wells of a 96-well microtiter plate and the plates were incubated further at 37 °C for 24 h. Wells containing bacterial strains and TSB were used as negative controls. The biofilm viability was measured following the previous evaluation protocol [103,104].

#### 4.3.6. Scanning Electron Microscopy of Biofilms

The cell morphology of the tested model strains’ biofilms after treatment with H-AuNFs and R-AuNFs was assessed with SEM according to a method described by Rafaque et al. [105] with minor modifications. Briefly, biofilms were allowed to grow in a 6-well plate as described above (see Section 4.3.4). For SEM analysis, each sample was fixed with 2.5% glutaraldehyde at 4 °C for at least 4 h, and then samples were washed three times with PBS (pH 7.4). Next, the samples were dehydrated in increasing concentrations (30%, 50%, 70%, 90%, and 2 × 100 % [*v*/*v*]), for 10 min at each concentration. Finally, samples were air-dried overnight and coated with a gold-palladium using an ion sputter coater (ZEISS, Jena, Germany). Each sample was examined with SEM equipment (ZEISS, Model: GEMINI 500, Jena, Germany).

#### 4.3.7. Statistical Analysis

The conformity of the data to the normal distribution was evaluated with the Shapiro–Wilk test. Differences between groups (H-AuNFs and R-AuNFs) in terms of optical density were compared with the Mann–Whitney U test. The variation between dilutions was presented by bar graph. IBM Spss 25 package program was used in the analysis and *p* < 0.05 was considered statistically significant.

## 5. Conclusions

In this study, successfully synthesizing AuNFs using a green approach without involving toxic chemicals demonstrated that rosemary and immortelle plant extracts served as good reducer and stabilizer agents during AgNPs synthesis. AuNFs manifested size-dependent antibacterial and antibiofilm activity. In line with this observation, the small-sized H-AuNFs exhibited superior inhibition against model strains compared to that of R-AuNFs due to greater interaction with bacterial cells. Thus, synthesized AuNFs in this study could be potential agents for controlling bacteria that are antibiotic resistant and grow on biofilms without developing resistance in bacteria in the medicinal and industrial areas. However, further studies are required to understand the molecular mechanisms, signal pathways, and in vivo antibacterial effects of these AuNFs.

## Figures and Tables

**Table 1:** The MIC and MBC values of AuNFs against tested bacterial strains.

Bacteria	MIC (µg/mL)		MBC (µg/mL)		Tolerance (MBC/MIC Ratio)	
	H-AuNFs	R-AuNFs	H-AuNFs	R-AuNFs	H-AuNFs	R-AuNFs
*S. aureus*	20	160	20	320	1	2
*S. epidermidis*	20	40	20	80	1	2
*E. coli*	20	40	20	80	1	2

**Table 2:** The biofilm inhibition and disruption rate of AuNFs against tested bacterial strains at various concentrations.

AuNFs	Concentration (µg/mL)	Biofilm Inhibition Rate (%)			Biofilm Disruption Rate (%)		
		*S. aureus*	*S. epidermidis*	*E. coli*	*S. aureus*	*S. epidermidis*	*E. coli*
H-AuNFs	320	96.0	95.8	95.8	91.0	81.5	77.5
	160	91.6	88.3	87.0	84.4	72.6	66.7
	80	90.0	86.2	84.6	82.2	65.0	64.6
	40	87.2	84.9	81.4	80.9	61.9	62.0
	20	84.9	82.7	79.7	79.7	59.8	59.3
R-AuNFs	320	91.2	89.1	85.2	82.7	73.0	70.1
	160	86.9	85.0	79.5	79.0	69.9	64.1
	80	84.2	83.1	78.4	76.6	66.4	63.0
	40	72.8	79.5	77.7	75.4	59.7	62.3
	20	69.6	72.5	72.8	74.6	55.5	57.7

## Data Availability

The data presented in this study are available upon reasonable request from the corresponding author.

## Supplementary Materials

The following supporting information can be downloaded at: https://www.mdpi.com/article/10.3390/antibiotics11111466/s1. Figure S1: Scanning electron microscopy (SEM) imaging of S. epidermidis, treated with H-AuNFs and R-AuNFs in biofilms formed after 24 h of incubation. (A,B) Control (Untreated); (C) treated with 320 µg/mL of H-AuNFs; (D) treated with 20 µg/mL of H-AuNFs; (E) treated with 320 µg/mL of R-AuNFs; (F); treated with 20 µg/mL of R-AuNFs; Figure S2: Scanning electron microscopy (SEM) imaging of *S. aureus*, treated with H-AuNFs and R-AuNFs in biofilms formed after 24 h of incubation. (A,B) Control (Untreated); (C) treated with 320 µg/mL of H-AuNFs; (D) treated with 20 µg/mL of H-AuNFs; (E) treated with 320 µg/mL of R-AuNFs; (F) treated with 20 µg/mL of R-AuNFs; Figure S3: Scanning electron microscopy (SEM) imaging of *E. coli*, treated with H-AuNFs and R-AuNFs in biofilms formed after 24 h of incubation. (A,B) Control (Untreated); (C) treated with 320 µg/mL of R-AuNFs; (D) treated with 20 µg/mL of R-AuNFs; (E) treated with 320 µg/mL of H-AuNFs; (F); treated with 20 µg/mL of H-AuNFs.
