# Supplementary material for: Green Synthesis of Gold Nanoflowers Using Rosmarinus officinalis and Helichrysum italicum Extracts: Comparative Studies of Their Antimicrobial and Antibiofilm Activities
Source: Antibiotics (Basel). 2022 Oct 25;11(11):1466. doi: 10.3390/antibiotics11111466 (PMC9686685; doi:10.3390/antibiotics11111466)
Supplement: Supplementary file 1 [file antibiotics-11-01466-s001.zip › antibiotics-1942893-supplementary.pdf]

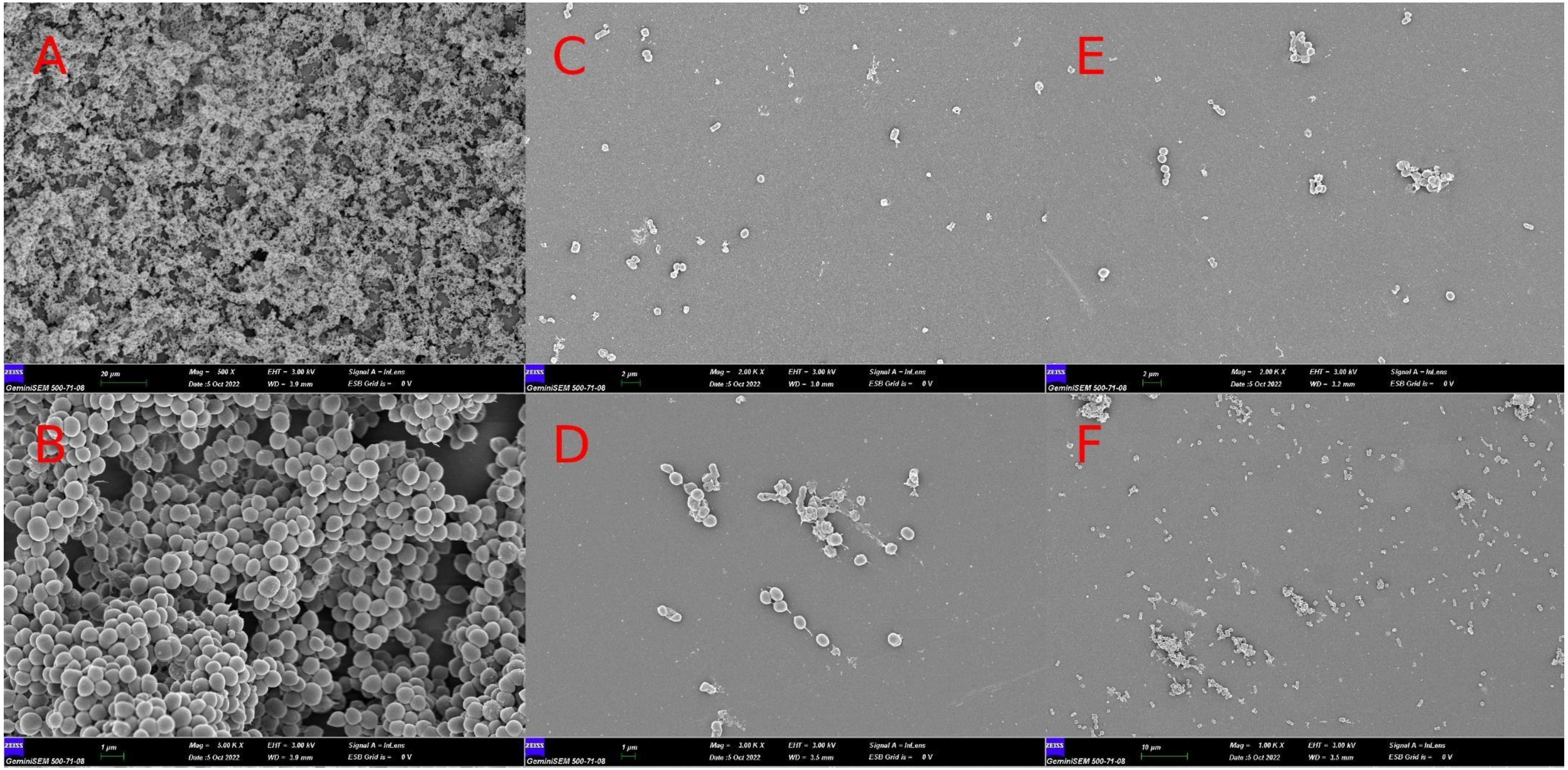

Figure S1. Scanning electron microscopy (SEM) imaging of *S. epidermidis*, treated with H-AuNFs and R-AuNFs in biofilms formed after 24 h of incubation. (**A**, **B**) Control (Untreated); (**C**) treated with 320 µg/mL of H-AuNFs; (**D**) treated with 20 µg/mL of H-AuNFs; (**E**) treated with 320 µg/mL of R-AuNFs; (**F**); treated with 20 µg/mL of R-AuNFs

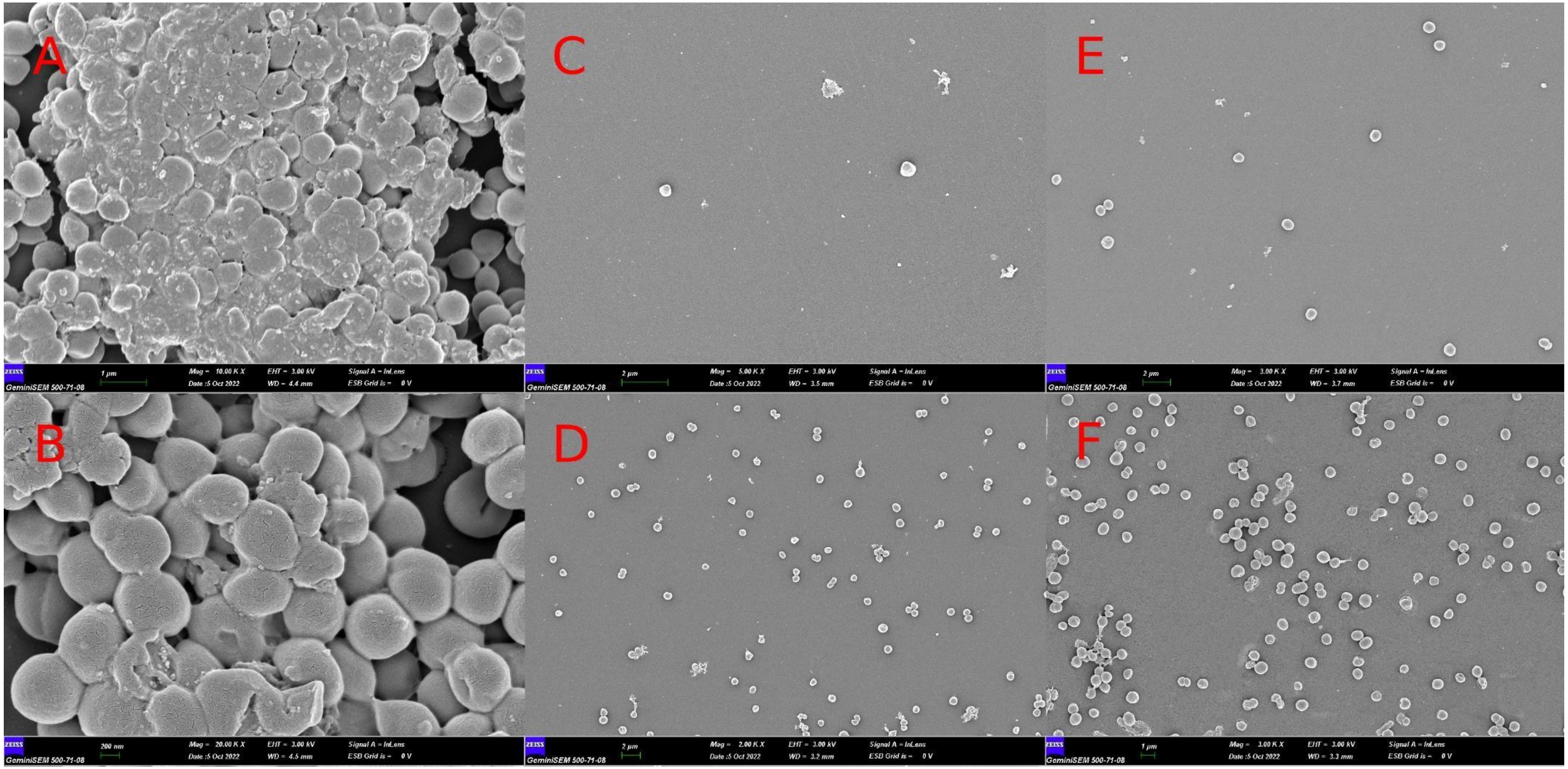

Figure S2. Scanning electron microscopy (SEM) imaging of *S. aureus*, treated with H-AuNFs and R-AuNFs in biofilms formed after 24 h of incubation. (**A**, **B**) Control (Untreated); (**C**) treated with 320 μg/mL of H-AuNFs; (**D**) treated with 20 μg/mL of H-AuNFs; (**E**) treated with 320 μg/mL of R-AuNFs; (**F**); treated with 20 μg/mL of R-AuNFs

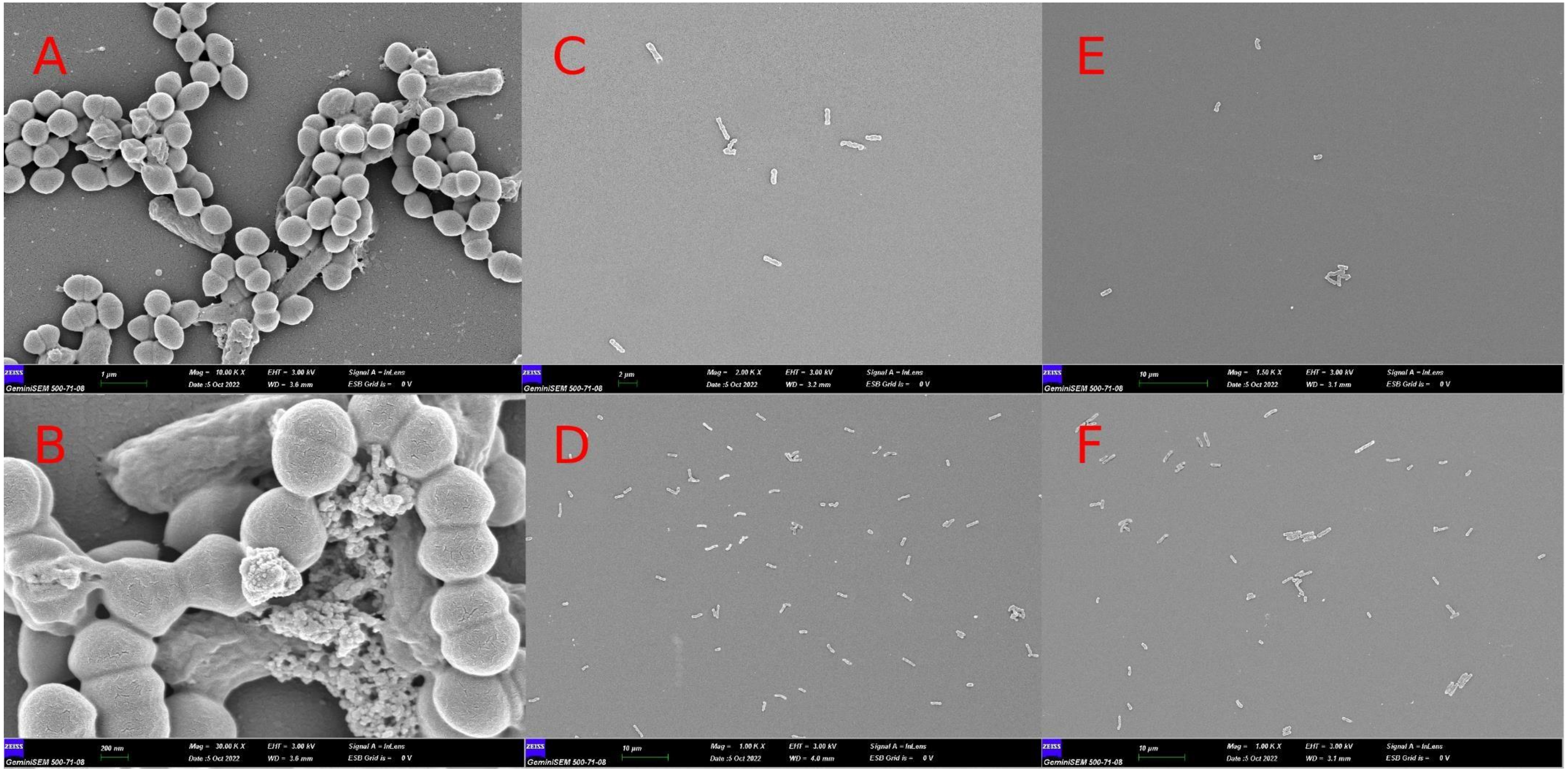

Figure S3. Scanning electron microscopy (SEM) imaging of *E. coli*, treated with H-AuNFs and R-AuNFs in biofilms formed after 24 h of incubation. (**A**, **B**) Control (Untreated); (**C**) treated with 320 µg/mL of R -AuNFs; (**D**) treated with 20 µg/mL of R-AuNFs; (**E**) treated with 320 µg/mL of H-AuNFs; (**F**); treated with 20 µg/mL of H-AuNFs
